# Supplementary material for: In situ observation of a stepwise [2 + 2] photocycloaddition process using fluorescence spectroscopy
Source: Nat Commun. 2023 Nov 27;14:7766. doi: 10.1038/s41467-023-42604-9 (PMC10682429; doi:10.1038/s41467-023-42604-9)
Supplement: Supplementary file 1 — Supplementary Information [file 41467_2023_42604_MOESM1_ESM.pdf]

Supplementary information

In situ observation of a stepwise [2+2] photocycloaddition process by fluorescence spectroscopy

Meng-Fan Wang[1,2], Yun-Hu Deng[1], Jia-Hui Gu[1], Yong-Yong Cao[3], Qi Liu[1]*, Pierre Braunstein[4], Jian-Ping Lang[1,2]*

[1]College of Chemistry, Chemical Engineering and Materials Science, Soochow University, Suzhou 215123, Jiangsu, People's Republic of China
[2]State Key Laboratory of Organometallic Chemistry, Shanghai Institute of Organic Chemistry, Chinese Academy of Sciences, Shanghai 200032, People's Republic of China
[3]College of Biological, Chemical Science and Engineering, Jiaxing University, Jiaxing 314001, Zhejiang, People's Republic of China
[4]Institut de Chimie (UMR 7177 CNRS), Université de Strasbourg, 4 rue Blaise Pascal - CS 90032, 67081 Strasbourg, France

Correspondence and requests for materials should be addressed to Q.L. (email: qi.liu@suda.edu.cn) or to J.P.L. (email: jplang@suda.edu.cn)

Contents

I. Supplementary Notes 5

Materials 5

Characterization 5

Explanations of any A- or B-level alerts 6

II. Supplementary Methods 7

Synthetic procedures 7

Kinetic analysis of each step 8

III. Supplementary Figures and Tables 10

Supplementary Figure 1 | Coordination polymer structure of CP1. a Representation of the coordination environment of the Cd(II) ions in **CP1**. **b** View of a section of the one-dimensional (1D) chain structure of **CP1**. The distance between the parallel C=C groups is 3.82 Å (orange dashed lines), while the separation between the crossed C=C pairs in **CP1** is 3.69 Å (orange dashed lines). **c** View of four adjacent 1D chains 10

Supplementary Figure 2 | Verification of the transformation from CP1 to CP1-2β by ^{19}F NMR spectra. The ^{19}F NMR spectra of the sample obtained from **CP1** irradiated under UV light (λ = 365 nm) at room temperature (DMSO-d_6). 10

Supplementary Figure 3 | Verification of the transformation from CP1 to CP1-1 by ^{19}F NMR spectra. The ^{19}F NMR spectra of the sample obtained from **CP1** irradiated under UV light (λ = 365 nm) at -50 °C (DMSO-d_6). 11

Supplementary Figure 4 | Verification of the transformation from CP1-1 to CP1-2β by ^{19}F NMR spectra. The ^{19}F NMR spectra of the sample obtained by irradiation of **CP1-1** under UV light (λ = 365 nm) at room temperature (DMSO-d_6). 11

Supplementary Figure 5 | UV-vis adsorption spectra. Solid state UV-vis adsorption spectra of **CP1** irradiated under UV light (λ = 365 nm) at room temperature for different periods of time...... 12

Supplementary Figure 6 | PXRD patterns characterization. a The PXRD patterns of **CP1**: simulated, as synthesized and after being suspended in ethanol for 24 h. **b** The PXRD patterns of **CP1-1**: simulated, as synthesized and after being suspended in ethanol for 24 h. **c** The PXRD patterns of **CP1-2β**: simulated, as synthesized and after being suspended in ethanol for 24 h. 12

Supplementary Figure 7 | Fluorescence properties. Solid state fluorescence excitation and emission spectra of F-1,3-bpeb, **CP1**, **CP1-1** and **CP1-2β**. 13

Supplementary Figure 8 | Fluorescence photographs. The powder **CP1** was irradiated at room temperature (λ = 365 nm). 13

Supplementary Figure 9 | Fluorescence lifetime analysis. The fluorescence lifetime (*τ*) of **CP1**, **CP1-1** and **CP1-2β** at room temperature. 14

Supplementary Figure 10 | Conversion obtained from ^{19}F NMR analysis. The conversion (mole %) calculated from ^{19}F NMR data of **CP1** under UV light (λ = 365 nm) irradiation at -50 °C and **CP1-1** under UV light (λ = 365 nm) irradiation at 25 °C as a function of time. 14

Supplementary Figure 11 | Kinetics analysis of each step calculated from ^{19}F NMR data sets. a The plot of c_0-c_t versus time indicating zero-order behavior in the transformation from **CP1** to **CP1-1**. c_0 and c_t represents the mole fraction calculated from ^{19}F NMR data sets of **CP1** before and at any irradiation time at 365 nm and -50 °C, respectively. *k* is the rate constant. **b** The plot of ln(-ln(1-*y*)) versus ln(time) fitted by JMAK model to study photochemical reaction kinetics of the transformation from **CP1-1** to **CP1-2β**, where *y* is the mole fraction of the photoproduct calculated from ^{19}F NMR data sets of **CP1-1** irradiated at 365 nm and 25 °C. *k* is the rate constant, and *n* is the dimensionality of growth (Avrami exponent). 15

Supplementary Figure 12 | Conversion obtained from fluorescence intensity analysis. The conversion (mole %) calculated from fluorescence intensity data sets of **CP1** under UV light (λ = 365 nm) irradiation at -50 °C and **CP1-1** under UV light (λ = 365 nm) irradiation at 25 °C as a function of time. 15

Supplementary Figure 13 | Correlation of the conversion obtained from NMR and fluorescence data sets. a The relationship between the conversion (mole %) calculated from NMR and fluorescence (FL) data sets for the transformation process from **CP1** to **CP1-1**. Equation of the linear correlation: y = 0.935x, R-squared = 0.991. **b** The relationship between the conversion (mole %) calculated from NMR and fluorescence (FL) data sets for the transformation process from **CP1-1** to **CP1-2β**. Equation of the linear correlation: y = 0.989x, R-squared = 0.941. 16

Supplementary Table 1 | Fluorescence quantum yields of **CP1**, **CP1-1** and **CP1-2β** under excitation at different wavelengths.[a] 16

Supplementary Figure 14 | Reliability examination. a Continues sections of LSCM images in the timeseries scan of **CP1** crystal under 405 nm laser for 1 h, scale bars are 50 μm. **b** Quantified luminescence intensities of **CP1** after different times. 17

Supplementary Figure 15 | LSCM images in the z-stack scan of the CP1 crystal. a Continuous sections of LSCM images in the z-stack scan of the **CP1** crystal, scale bars are 50 μm. **b** 3D

reconstitution of LSCM of **CP1** based on continues sections of total 427 slices, scale bar is 50 μm. 18

Supplementary Figure 16 | LSCM images of CP1. The LSCM images of **CP1** irradiated under UV light for some time interval (t = 0, 1 min, 3 min, 10 min, 20 min, 40 min and 60 min) at different slices, scale bars are 50 μm. Z represents the number of slices, where each slice is 100 nm thick... 19

Supplementary Figure 17 | ESR spectra. The solid-state ESR spectra of **CP1** after irradiation under UV light (λ = 360 nm) for 2 min and 5 min. 20

Supplementary Figure 18 | Molecular interaction analysis. The molecular interactions of (**a**) **CP1**, (**b**) **CP1-1** and (**c**) **CP1-2β**. 20

Supplementary Figure 19 | Intermolecular interaction analysis. Hirshfeld surface analysis (mapped over d_{norm}), and proportions of intermolecular C···C, C···H, halogen···H, and other interactions to the total intermolecular interactions based on their crystal structures. 20

Supplementary Figure 20 | Theoretical calculation. Frontier molecular orbitals of optimized ground-state geometries of **CP1**, **CP1-1** and **CP1-2β** calculated by the sTDDFT method at the PBE0 D3 def2-TZVP level, ORCA 5.0.3 package. E_{Gap} represents calculated energy gap. 21

IV. Supplementary references 22

I. Supplementary Notes

Materials

All reagents were obtained commercially and used without further purification. 1,3-dibromo-5-fluorobenzene (Adamas, 98%), 4-vinylpyridine (Aladdin, 96%, stabilized), K_2CO_3 (Aladdin, 98%), $(PPh_3)_2PdCl_2$ (Adamas, 98%), 3,5-dibromobenzoic acid (TCI, 97%), sodium hydroxide (Aladdin, 96%), $3CdSO_4 \cdot 8H_2O$ (Aladdin, 99.0%) and anhydrous Na_2SO_4 (Aladdin, 99.0%) were used as received. Deoxygenated N,N-dimethylformamide (Aladdin, 99.8% Anhydrous) was degassed with N_2 and dried on molecular sieves before use. Concentrated HNO_3 was purchased from Shanghaihushi at F.W. 63.01% purity. DMSO-*d₆* (CIL, 99.9%) were used to collect nuclear magnetic resonance (NMR) spectra. All other solvents, dichloromethane, trichloromethane, and ethanol were purchased from Macklin at 99.8% purity and used without further purification. Nitrogen was purchased from Airgas at 99.9% purity.

Characterization

Powder X-ray diffraction (PXRD) patterns were acquired on a PANalytical X'Pert PRO MPD system (PW3040/60) using Cu Kα radiation (λ = 1.5406 Å) from 5° to 50° with a scanning step size of 0.02°. Elemental analyses (C, H, N) were performed using a PE 2400 II elemental analyzer. The NMR spectra were recorded at ambient temperature on a Bruker AVANCEШ HD-400M spectrometer. ^1H NMR and ^{19}F NMR chemical shifts were referenced to the solvent signal in $CDCl_3$ or DMSO-d_6. Chemical shifts are reported in parts per million (ppm) and referenced with TMS for ^1H NMR and $CFCl_3$ for ^{19}F NMR. Solid-state UV-vis absorption spectra were recorded on a Varian Cary-50 UV-vis spectrophotometer with an integrating sphere at room temperature in the range of 200 - 800 nm. The electron spin (paramagnetic) resonance (ESR/EPR) spectra were obtained with an JES-X320 electron spin resonance spectrometer operating at the X-band (frequency 9.148 GHz) for samples sealed inside a 4 mm thick quartz capillary, with irradiation by a Xe light (500 W, equipped with a filter ≤360 nm). Photoluminescence spectra and quantum yields were obtained on a HORIBA PTI QuantaMaster40 Spectrofluorometer and lifetimes were measured on a FLS980. Luminescence imaging was performed with a Leica TCS SP5 II confocal laser scanning microscope. The photo-irradiation experiments were conducted with a LED lamp NBT-LED4 (λ = 365 nm, 2 W).

Explanations of any A- or B-level alerts

The crystal used has cracks in this reaction, and thus the poor single-crystal quality of **CP1-2β** inevitably led to these B-level mistake in the Checkcif Report (PLAT342_ALERT_3_B Low Bond Precision on C-C Bonds .... 0.02785 Ang; PLAT601_ALERT_2_B Unit Cell Contains Solvent Accessible VOIDS of . 103 Ang**3).

II. Supplementary Methods

Synthetic procedures

Synthesis of 4,4'-(5-fluoro-1,3-phenylene)bis(ethene-2,1-diyl))dipyridine (F-1,3-bpeb). The ligand 4,4'-(5-fluoro-1,3-phenylene)bis(ethene-2,1-diyl))dipyridine (F-1,3-bpeb) was prepared according to the literature methods.[1]

The synthesis of F-1,3-bpeb ligand was based on the Heck reaction and using standard Schlenk techniques. To a 200 mL Schlenk tube involving 1,3-dibromo-5-fluorobenzene (2.52 g, 0.01 mol), 4-vinylpyridine (2.31 g, 0.022 mol), and K_2CO_3 (2.76 g, 0.02 mol) was added $(PPh_3)_2PdCl_2$ (0.084 g, 0.12 mmol). The tube was degassed under vacuum and then backfilled with N_2 for three times. Deoxygenated N,N-dimethylformamide (DMF, 50 mL) was added into the tube under N_2, the tube sealed, and the mixture heated to 120 °C with stirring for 2 days. After cooling to ambient temperature, the solid dark mass was dissolved in CH_2C1_2 (100 mL), extracted thoroughly with water (3 × 50 mL), and dried over anhydrous Na_2SO_4. The organic phase was concentrated under vacuum to give F-1,3-bpeb ligand as a light yellow powder. Yield: 2.55 g (84.4%). ^{1}H NMR (400 MHz, DMSO-d_6): δ 8.59 (d, ^3J(H,H) = 6.0 Hz, 4 H; pyridyl), 7.79 (s, 1 H), 7.59 (d, ^3J(H,H) = 16.0 Hz, 2 H; CH=CH), 7.58 (d, ^3J(H,H) = 6.0 Hz, 4 H; pyridyl), 7.52 (d, ^3J(H,H) = 10.0 Hz, 2 H), 7.43 (d, ^3J(H,H) = 16.0 Hz, 2 H; CH=CH); ^{13}C NMR (100 MHz, DMSO-d_6): δ 164.07,161.66, 150.15, 143.78, 139.14, 131.50, 128.06, 122.28, 120.98, 113.29, 113.06; ^{19}F NMR (377 MHz, DMSO-d_6): δ -113.13 ppm; analysis (calcd., found for $C_{20}H_{15}FN_2$): C (79.45, 79.29), H (5.00, 5.10), N (9.27, 9.20).

Synthesis of coordination polymers. The coordination polymers $[Cd_2(F\text{-}1,3\text{-}bpeb)_2(3,5\text{-}DBB)_4]$ (**CP1**, 3,5-HDBB = 3,5-dibromobenzoic acid), $[Cd_2(1)(3,5\text{-}DBB)_4]$ **(CP1-1)** and $[Cd_4(2\beta)_2(3,5\text{-}DBB)_8]$ **(CP1-2β)** were synthesized according to the literature methods.[2]

CP1: A thick Pyrex tube was loaded with $3CdSO_4\cdot 8H_2O$ (25.0 mg, 0.092 mmol), F-1,3-bpeb (6.0 mg, 0.021 mmol), 3,5-HDBB (11.7 mg, 0.042 mmol), 1.5 mL of DMF/H_2O (v/v = 1:4) and one drop of concentrated HNO_3. The tube was sealed and heated at 140 °C for 5 h. It was then cooled to room temperature to form colorless crystals of **CP1**, which were collected by filtration, washed with EtOH and H_2O, and dried in air. Yield: 90% (based on F-1,3-bpeb). ^{1}H NMR (400 MHz, DMSO-d_6): δ 8.60 (d, ^3J(H,H) = 6.0 Hz, 4 H; pyridyl), 8.04 (s, 4 H), 7.95 (s, 2 H), 7.79 (s, 2 H), 7.60 (d, ^3J(H,H) = 16.0 Hz, 2 H; CH=CH), 7.59 (d, ^3J(H,H) = 6.0 Hz, 4 H; pyridyl), 7.52 (d, J = 10.0 Hz, 2 H), 7.44 (d, ^3J(H,H) = 16.0 Hz, 2 H; CH=CH); ^{19}F NMR (377 MHz, DMSO-d_6): δ -113.13 ppm; analysis (calcd., found for $C_{68}H_{42}Br_8CdF_2N_4O_8$): C (44.56, 44.64), H (2.31, 2.25), N (3.06, 3.18).

CP1-1: The as-synthesized crystals of **CP1** deposited on the quartz plate were placed in a long glass tube which was immersed in a low-temperature thermostatic reaction bath at -50 °C and irradiated with a LED lamp (365 nm, 2 W) for 10 min to form faint yellow crystals of **CP1-1** (100% yield based on **CP1**). ^{1}H NMR (400 MHz, DMSO-d_6): δ 8.50 (d, ^3J(H,H) = 6.0 Hz, 4 H; pyridyl), 8.39 (d, ^3J(H,H) = 6.0 Hz, 4 H; pyridyl), 8.04 (s, 8 H), 7.95 (s, 4 H), 7.48 (m, 8 H), 7.28 (d, ^3J(H,H) = 6.0 Hz, 4 H; pyridyl), 7.23 (d, ^3J(H,H) = 16.0 Hz, 2 H; CH=CH), 7.09 (d, J = 9.6 Hz, 2 H), 4.74 (d, J = 6.8 Hz, 2 H; CH-CH), 4.67 (d, J = 6.8 Hz, 2 H; CH-CH); ^{19}F NMR (377 MHz, DMSO-d_6): δ -113.82 ppm; analysis (calcd., found for $C_{68}H_{42}Br_8CdF_2N_4O_8$): C (44.56, 44.49), H (2.31, 2.41), N (3.06, 2.95).

CP1-2β: The as-synthesized crystals of **CP1** on the quartz plate were irradiated with a LED lamp (365 nm, 2 W) for 1 h at 25 °C or the as-synthesized crystals of **CP1-1** on the quartz pieces were irradiated with a LED lamp (365 nm, 2 W) for 35 min at 25 °C to form yellow crystals of **CP1-2β** (100% yield based on **CP1** or **CP1-1**). ^{1}H NMR (400 MHz, DMSO-d_6): δ 8.43 (m, 8 H; pyridyl), 8.04 (s, 8 H), 7.98 (s, 4 H), 7.32 (d, ^3J(H,H) = 6.0 Hz, 4 H; pyridyl), 7.27 (d, ^3J(H,H) = 6.0 Hz, 4 H; pyridyl), 7.17 (s, 2 H), 6.71 (d, J = 9.2 Hz, 2 H), 6.46 (d, J = 9.2 Hz, 2 H), 4.84 (m, 8 H; CH-CH); ^{19}F NMR (377 MHz, DMSO-d_6): δ -114.87 ppm; analysis (calcd., found for $C_{136}H_{84}Br_{16}Cd_2F_4N_8O_{16}$): C (44.56, 44.64), H (2.31, 2.43), N (3.06, 2.98).

Kinetic analysis of each step

In order to determine the kinetics of this reaction, we monitored the corresponding structural transformation upon UV irradiation by in situ time-dependent fluorescence spectra and ^{19}F NMR. The fitting of the conversion data calculated from fluorescence intensity versus irradiation time showed different kinetics for **CP1** to **CP1-1** and **CP1-1** to **CP1-2β**, respectively.

The kinetics of the transformation from **CP1-1** to **CP1-2β** was fitted by applying the Johnson-Mehl-Avrami-Kolmogorov (JMAK) model.[3-6] The JMAK kinetics is described by equation (1):

$$y = 1 - \mathrm{e}^{(kt)^n} \quad (1)$$

where y is the conversion (mole fraction) of the photoproduct formed in time t, k is the rate constant, and n is the dimensionality of growth (Avrami exponent).

The kinetics of the transformation from **CP1** to **CP1-1** was calculated by general equation[7,8] for zero-order reaction rate, which has been successfully applied previously to [2+2] photocycloaddition reactions. The kinetics is described by equation (2):

$$c_0 - c_\mathrm{t} = kt \quad (2)$$

where c_0 and c_t represent the conversion (mole fraction) of **CP1** before and at any irradiation time (*t*) at 365 nm and -50 °C, respectively. *k* is the rate constant.

III. Supplementary Figures and Tables

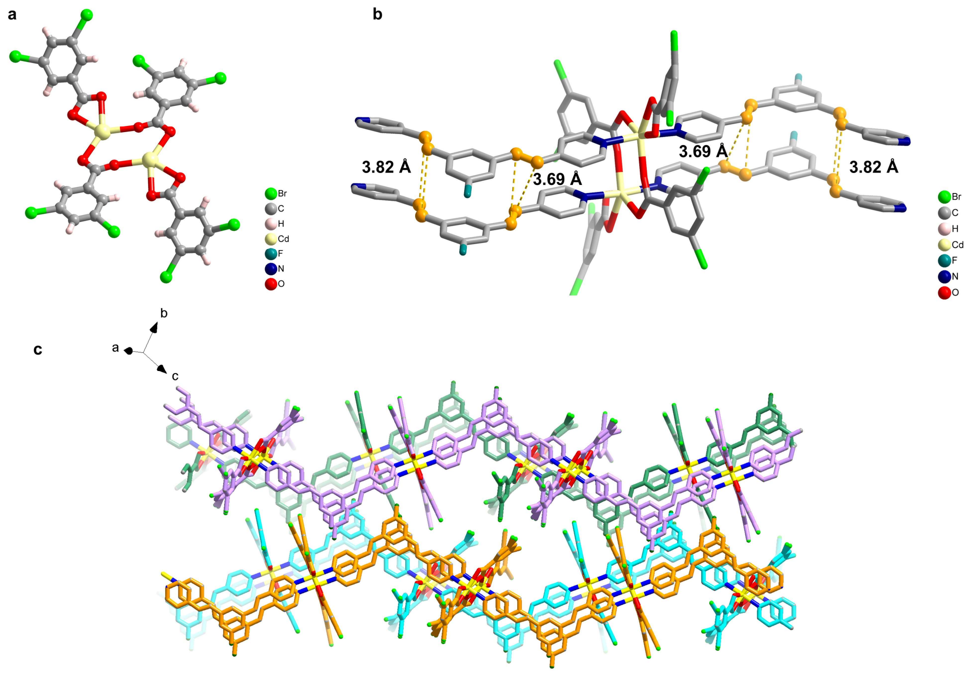

Supplementary Figure 1 | Coordination polymer structure of CP1. a Representation of the coordination environment of the Cd(II) ions in **CP1**. **b** View of a section of the one-dimensional (1D) chain structure of **CP1**. The distance between the parallel C=C groups is 3.82 Å (orange dashed lines), while the separation between the crossed C=C pairs in **CP1** is 3.69 Å (orange dashed lines). **c** View of four adjacent 1D chains.

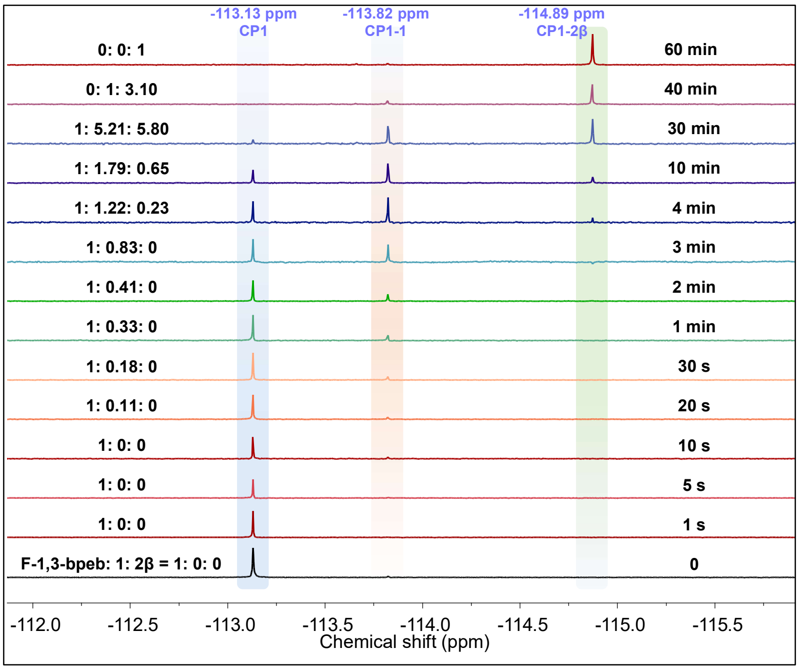

Supplementary Figure 2 | Verification of the transformation from CP1 to CP1-2β by ^{19}F NMR spectra. The ^{19}F NMR spectra of the sample obtained from **CP1** irradiated under UV light (λ = 365 nm) at room temperature (DMSO-d_6).

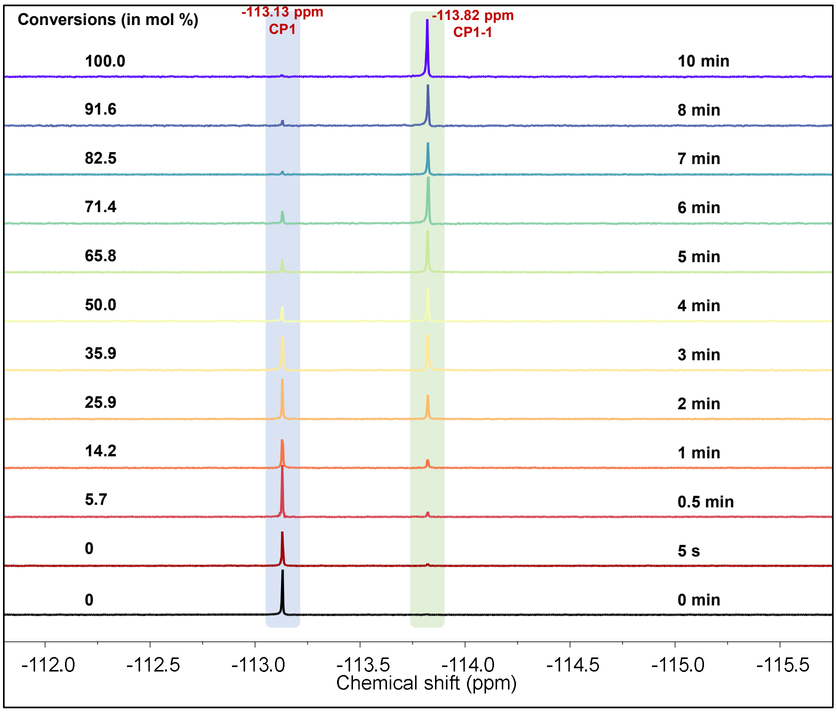

Supplementary Figure 3 | Verification of the transformation from CP1 to CP1-1 by ^{19}F NMR spectra. The ^{19}F NMR spectra of the sample obtained from **CP1** irradiated under UV light (λ = 365 nm) at -50 °C (DMSO-d_6).

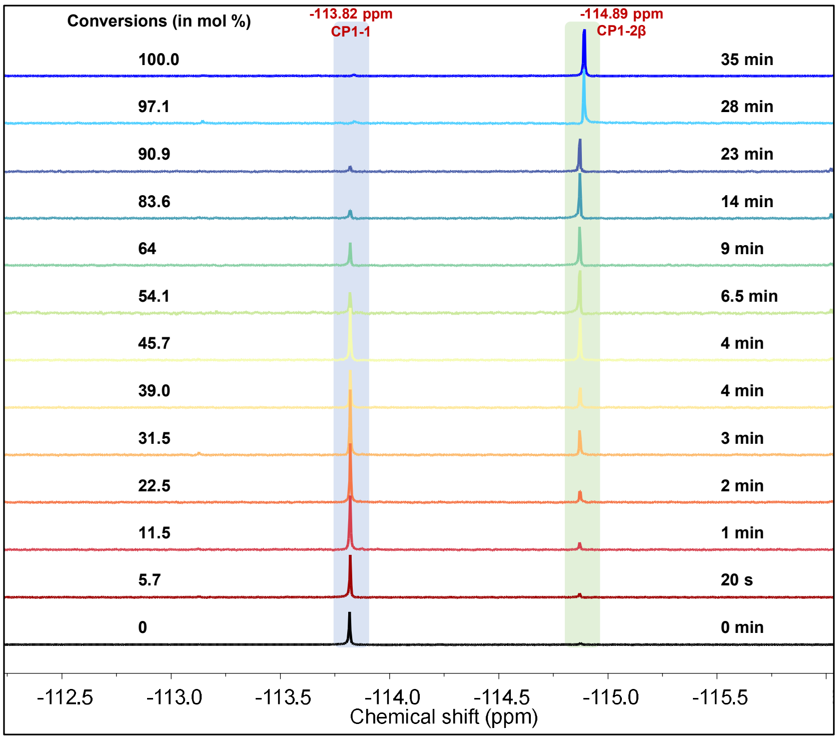

Supplementary Figure 4 | Verification of the transformation from CP1-1 to CP1-2β by ^{19}F NMR spectra. The ^{19}F NMR spectra of the sample obtained by irradiation of **CP1-1** under UV light (λ = 365 nm) at room temperature (DMSO-d_6).

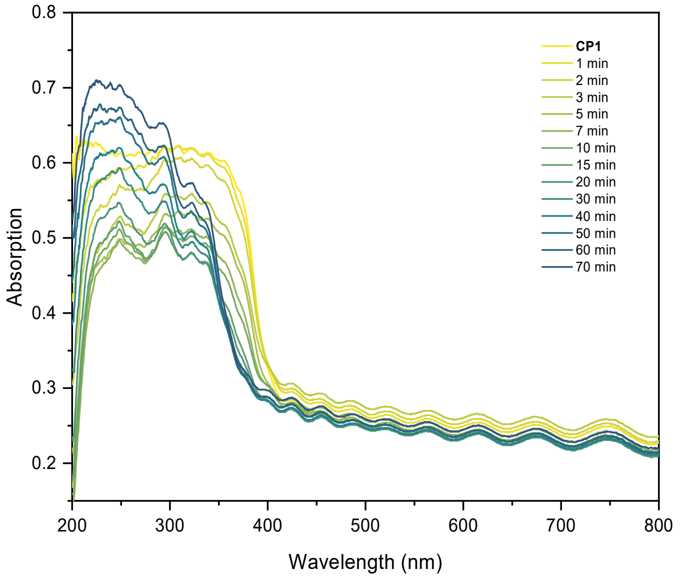

Supplementary Figure 5 | UV-vis adsorption spectra. Solid state UV-vis adsorption spectra of **CP1** irradiated under UV light (λ = 365 nm) at room temperature for different periods of time.

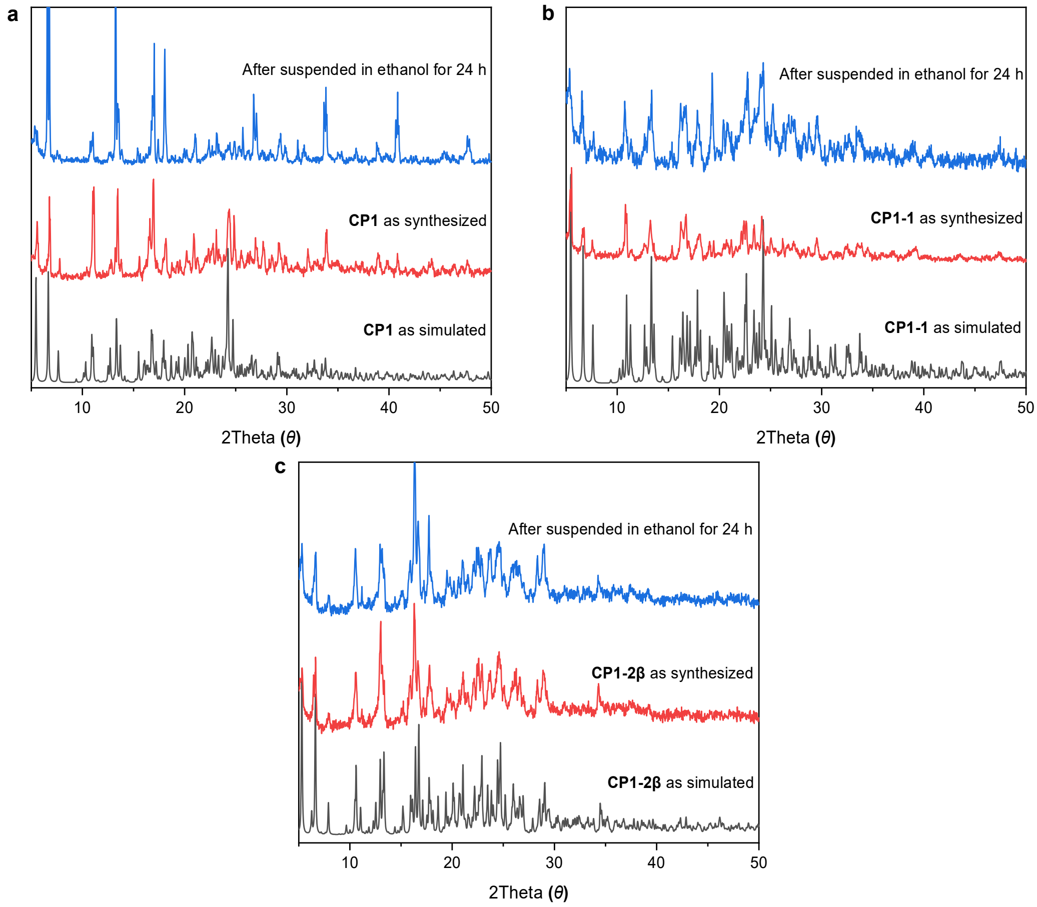

Supplementary Figure 6 | PXRD patterns characterization. a The PXRD patterns of **CP1**: simulated, as synthesized and after being suspended in ethanol for 24 h. **b** The PXRD patterns

of **CP1-1**: simulated, as synthesized and after being suspended in ethanol for 24 h. **c** The PXRD patterns of **CP1-2β**: simulated, as synthesized and after being suspended in ethanol for 24 h.

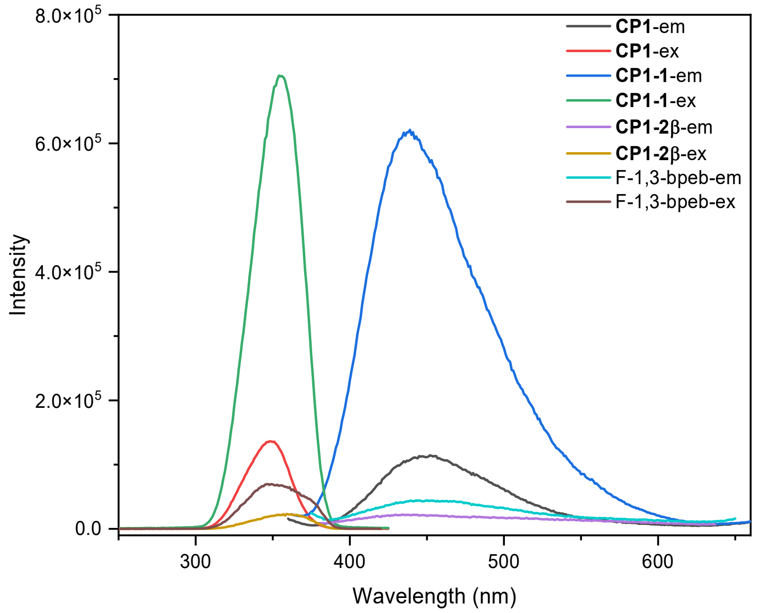

Supplementary Figure 7 | Fluorescence properties. Solid state fluorescence excitation and emission spectra of F-1,3-bpeb, **CP1**, **CP1-1** and **CP1-2β**.

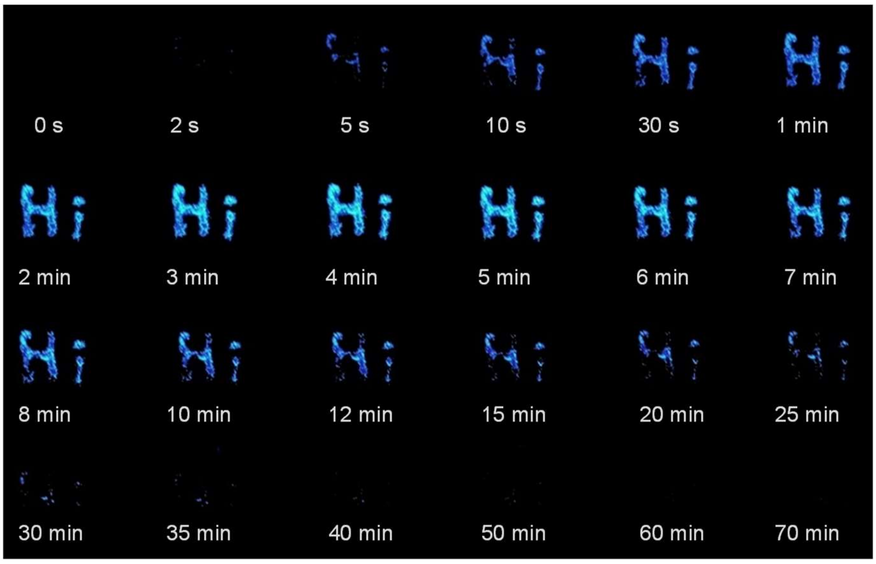

Supplementary Figure 8 | Fluorescence photographs. The powder **CP1** was irradiated at room temperature (λ = 365 nm).

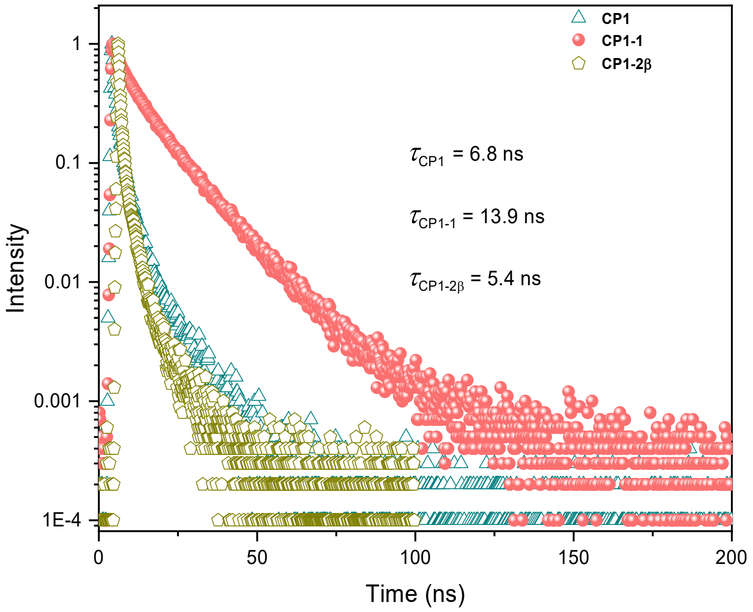

Supplementary Figure 9 | Fluorescence lifetime analysis. The fluorescence lifetime (τ) of **CP1**, **CP1-1** and **CP1-2β** at room temperature.

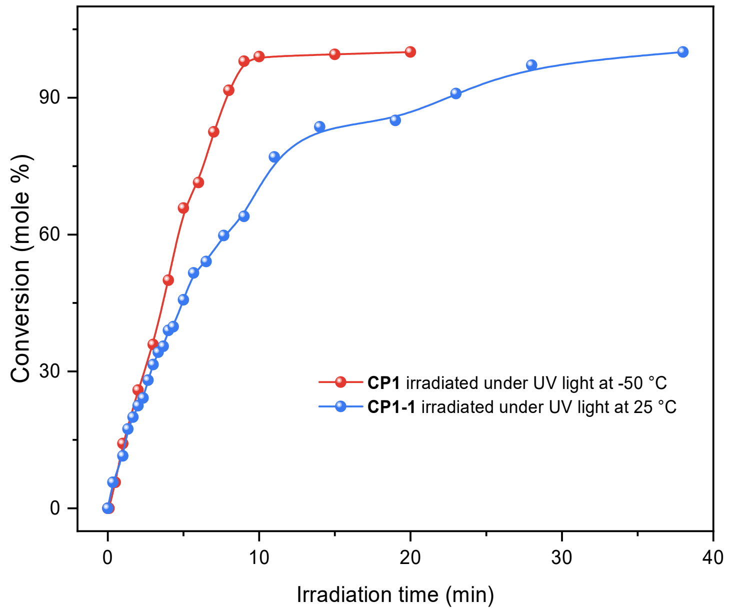

Supplementary Figure 10 | Conversion obtained from ^{19}F NMR analysis. The conversion (mole %) calculated from ^{19}F NMR data of **CP1** under UV light (λ = 365 nm) irradiation at -50 °C and **CP1-1** under UV light (λ = 365 nm) irradiation at 25 °C as a function of time.

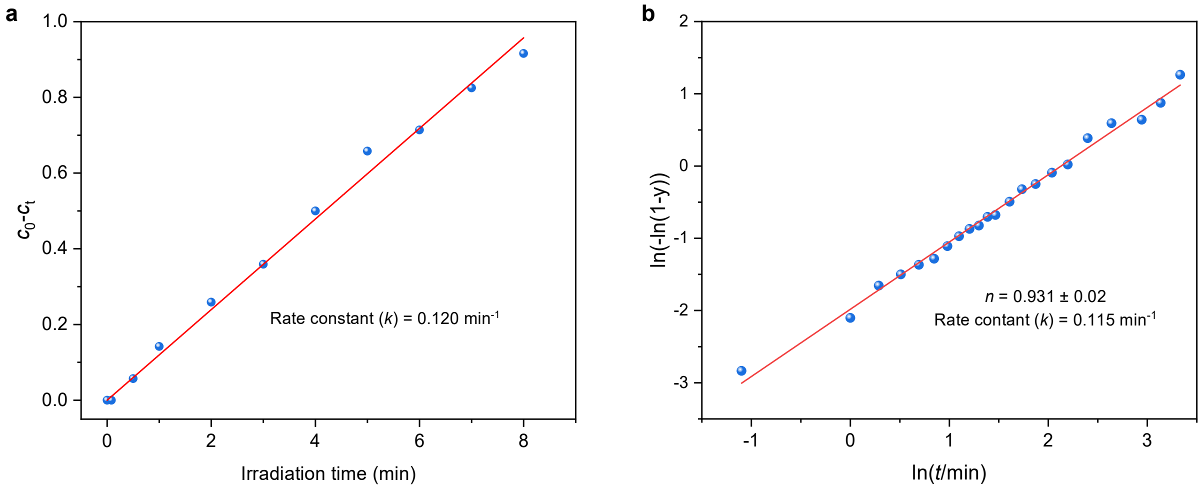

Supplementary Figure 11 | Kinetics analysis of each step calculated from ^{19}F NMR data sets. a The plot of c_0-c_t versus time indicating zero-order behavior in the transformation from **CP1** to **CP1-1**. c_0 and c_t represents the mole fraction calculated from ^{19}F NMR data sets of **CP1** before and at any irradiation time at 365 nm and -50 °C, respectively. k is the rate constant. **b** The plot of ln(-ln(1-y)) versus ln(time) fitted by JMAK model to study photochemical reaction kinetics of the transformation from **CP1-1** to **CP1-2β**, where y is the mole fraction of the photoproduct calculated from ^{19}F NMR data sets of **CP1-1** irradiated at 365 nm and 25 °C. k is the rate constant, and n is the dimensionality of growth (Avrami exponent).

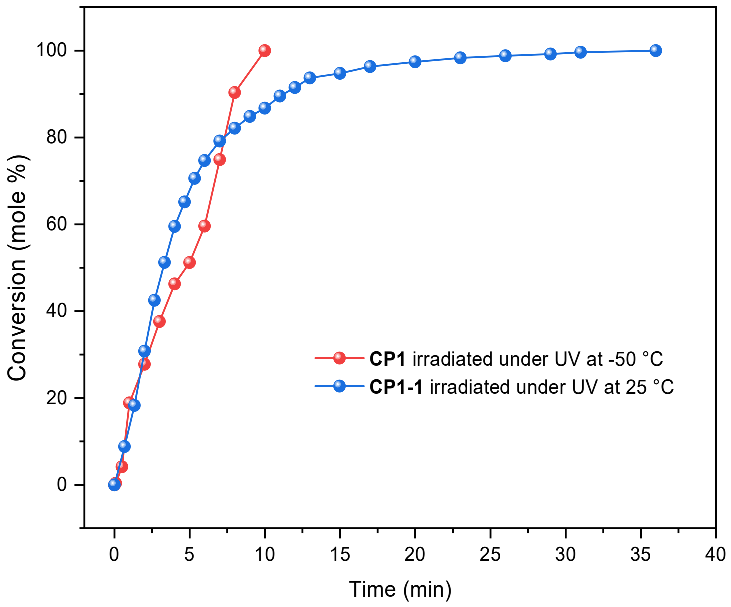

Supplementary Figure 12 | Conversion obtained from fluorescence intensity analysis. The conversion (mole %) calculated from fluorescence intensity data sets of **CP1** under UV light (λ = 365 nm) irradiation at -50 °C and **CP1-1** under UV light (λ = 365 nm) irradiation at 25 °C as a function of time.

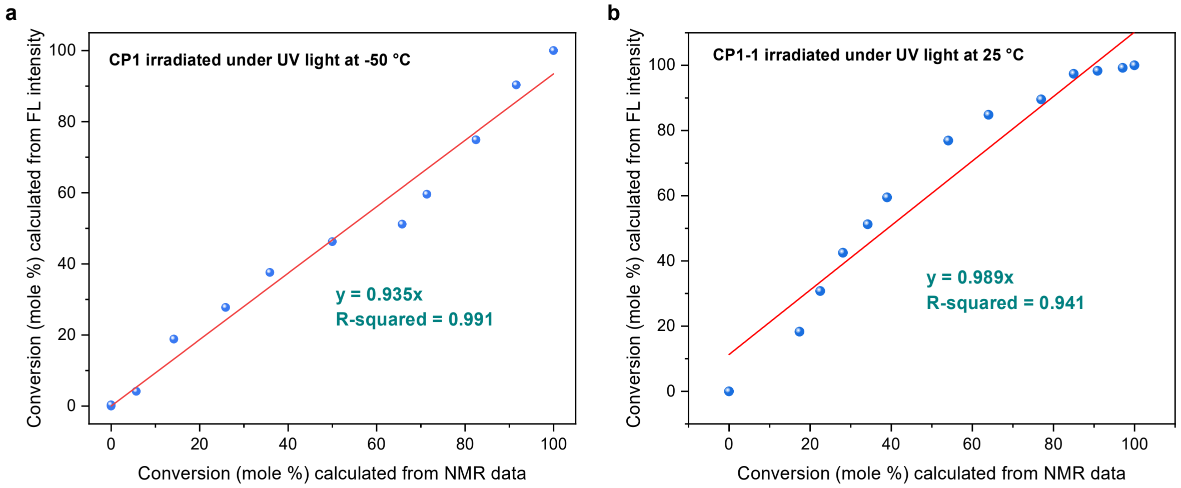

Supplementary Figure 13 | Correlation of the conversion obtained from NMR and fluorescence data sets. a The relationship between the conversion (mole %) calculated from NMR and fluorescence (FL) data sets for the transformation process from **CP1** to **CP1-1**. Equation of the linear correlation: y = 0.935x, R-squared = 0.991. **b** The relationship between the conversion (mole %) calculated from NMR and fluorescence (FL) data sets for the transformation process from **CP1-1** to **CP1-2β**. Equation of the linear correlation: y = 0.989x, R-squared = 0.941.

Supplementary Table 1 | Fluorescence quantum yields of **CP1**, **CP1-1** and **CP1-2β** under excitation at different wavelengths.[a]

	**CP1**	**CP1-1**	**CP1-2β**
λ_{ex} = 365 nm	7.8%	58.5%	1.2%
λ_{ex} = 405 nm	3.9%	24.9%	0.7%

[a]Fluorescence quantum yields of solid powder were measured by an integrating sphere.

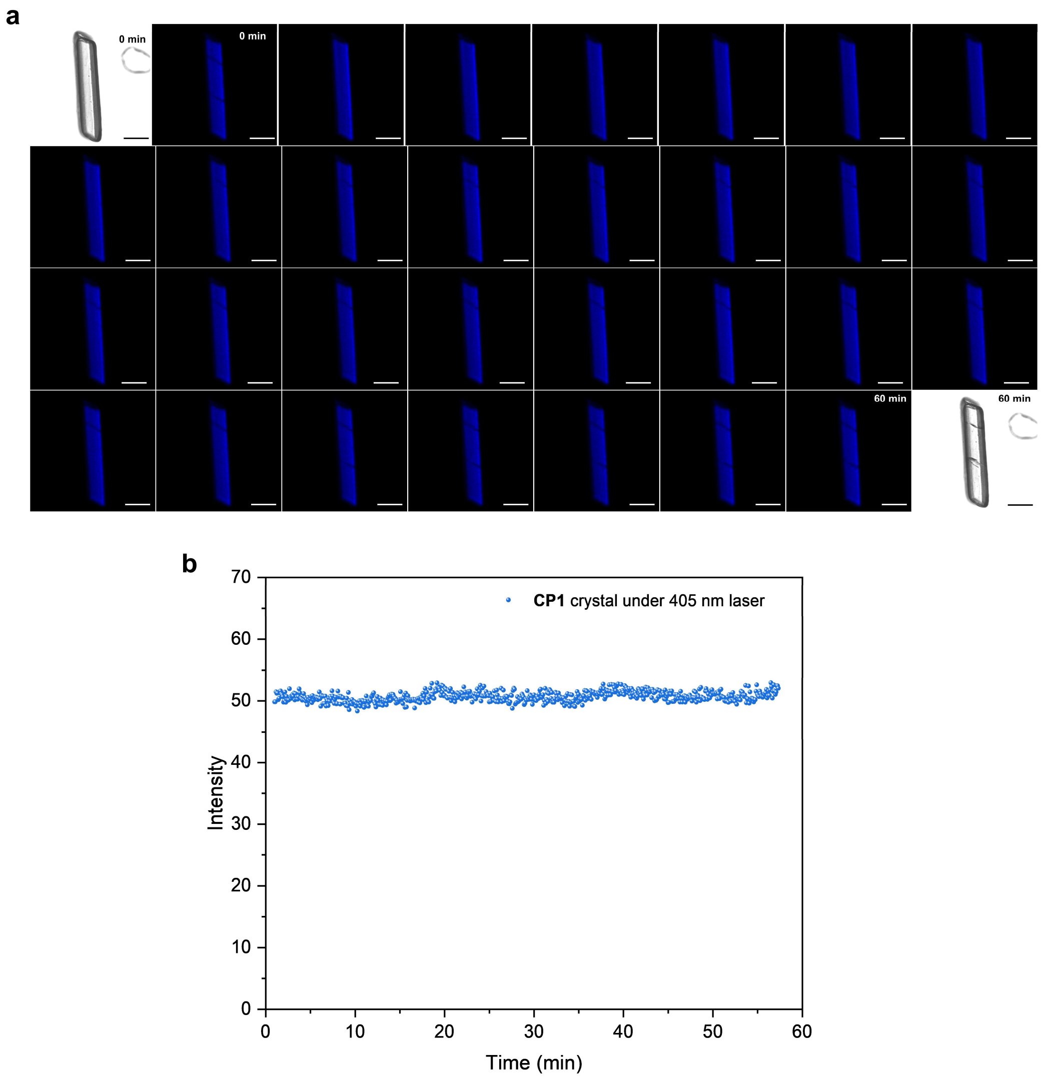

Supplementary Figure 14 | Reliability examination. a Continues sections of LSCM images in the timeseries scan of **CP1** crystal under 405 nm laser for 1 h, scale bars are 50 μm. **b** Quantified luminescence intensities of **CP1** after different times.

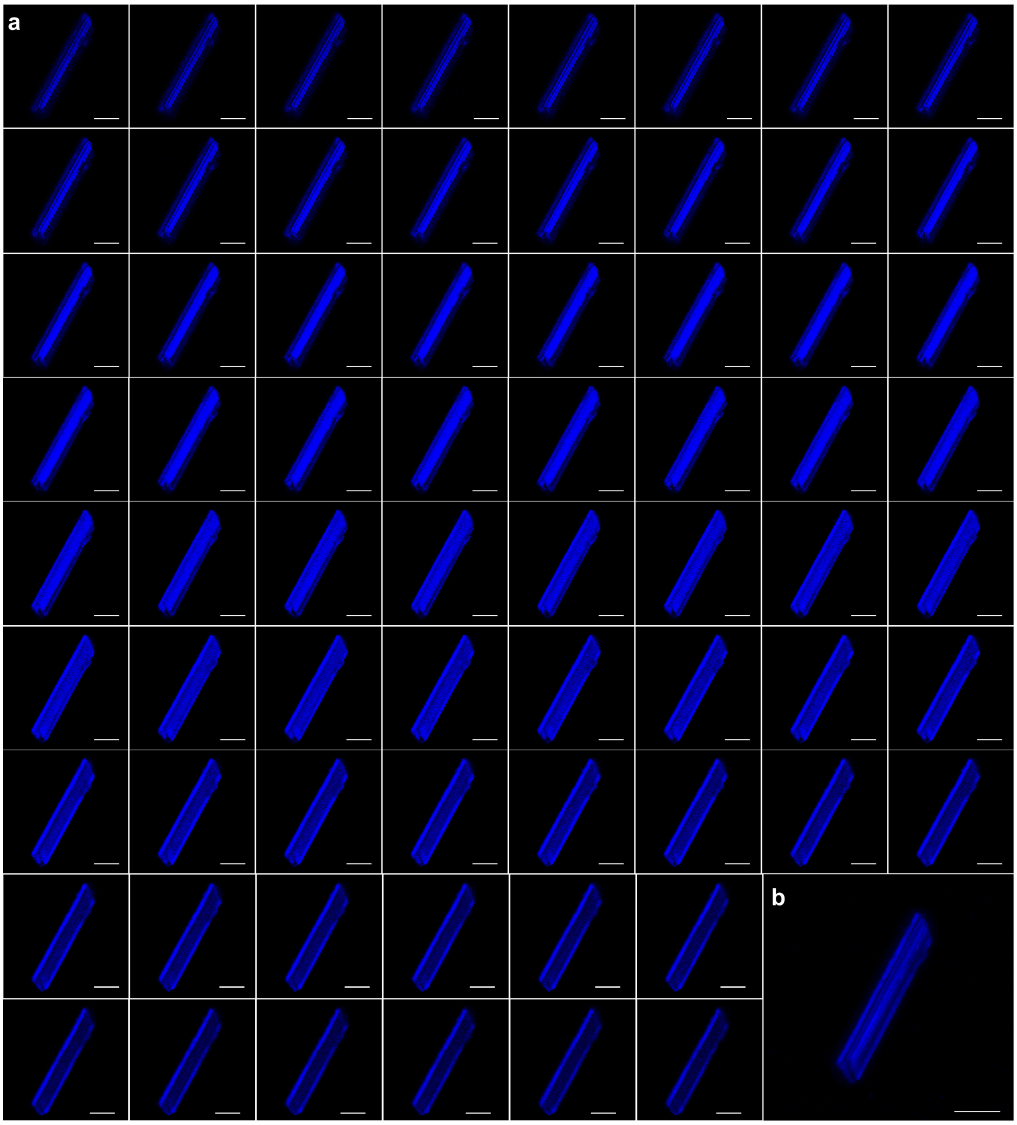

Supplementary Figure 15│LSCM images in the z-stack scan of the CP1 crystal. a Continuous sections of LSCM images in the z-stack scan of the **CP1** crystal, scale bars are 50 μm. **b** 3D reconstitution of LSCM of **CP1** based on continues sections of total 427 slices, scale bar is 50 μm.

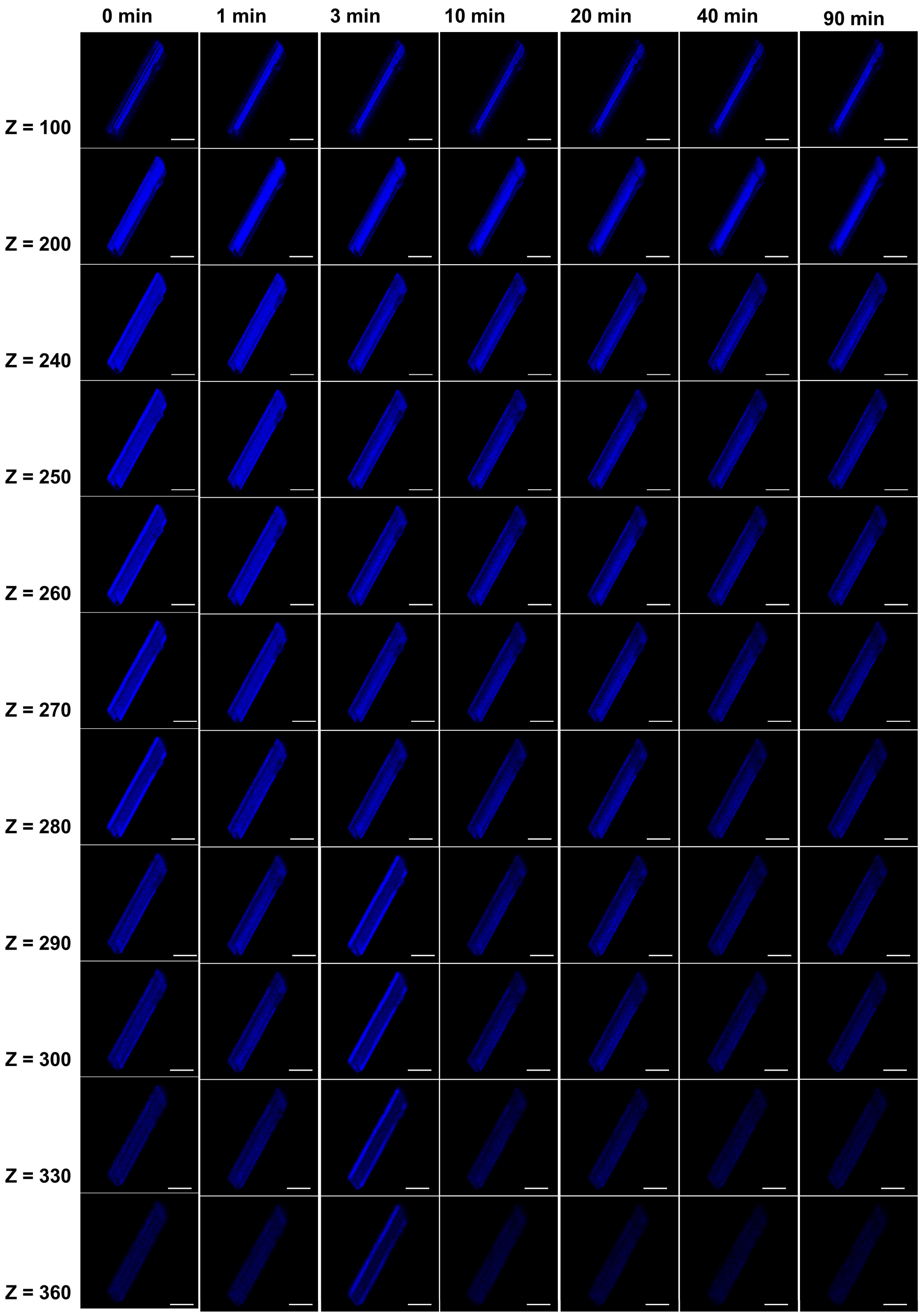

Supplementary Figure 16 | LSCM images of CP1. The LSCM images of **CP1** irradiated under UV light for some time interval (t = 0, 1 min, 3 min, 10 min, 20 min, 40 min and 60 min) at different slices, scale bars are 50 μm. Z represents the number of slices, where each slice is 100 nm thick.

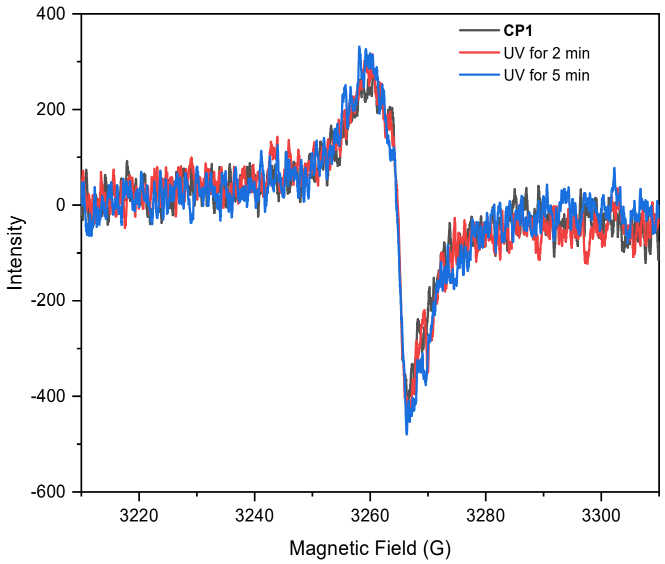

Supplementary Figure 17 | ESR spectra. The solid-state ESR spectra of **CP1** after irradiation under UV light (λ = 360 nm) for 2 min and 5 min.

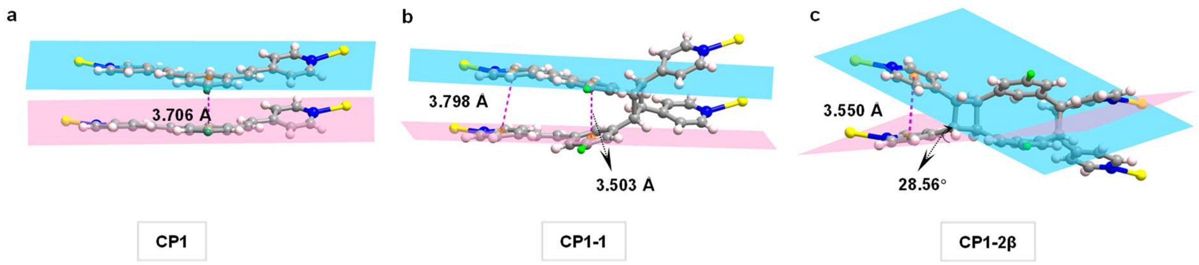

Supplementary Figure 18 | Molecular interaction analysis. The molecular interactions of (**a**) **CP1**, (**b**) **CP1-1** and (**c**) **CP1-2β**.

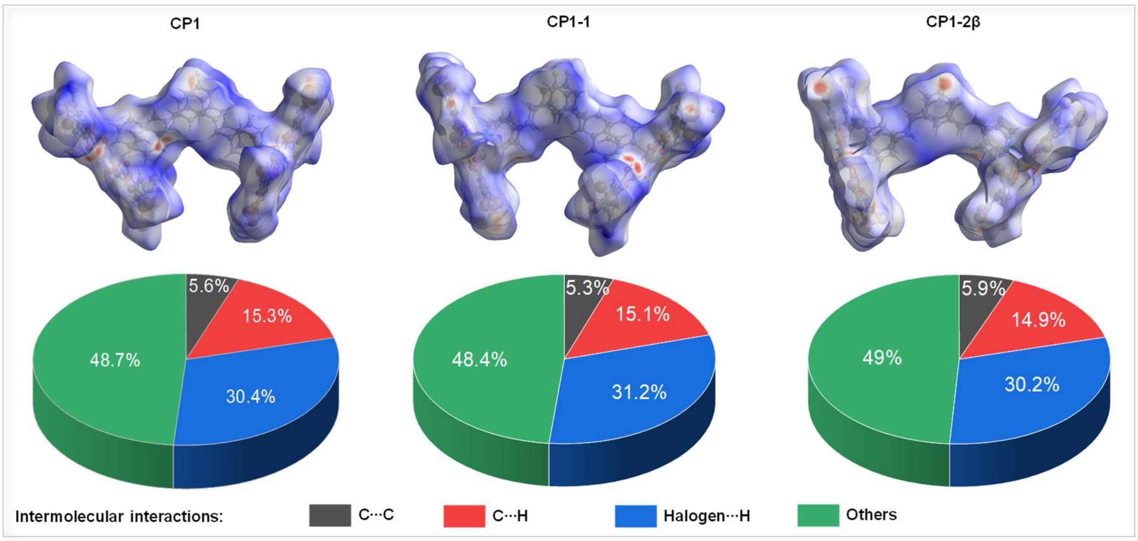

Supplementary Figure 19 | Intermolecular interaction analysis. Hirshfeld surface analysis (mapped over d_{norm}), and proportions of intermolecular C···C, C···H, halogen···H, and other interactions to the total intermolecular interactions based on their crystal structures.

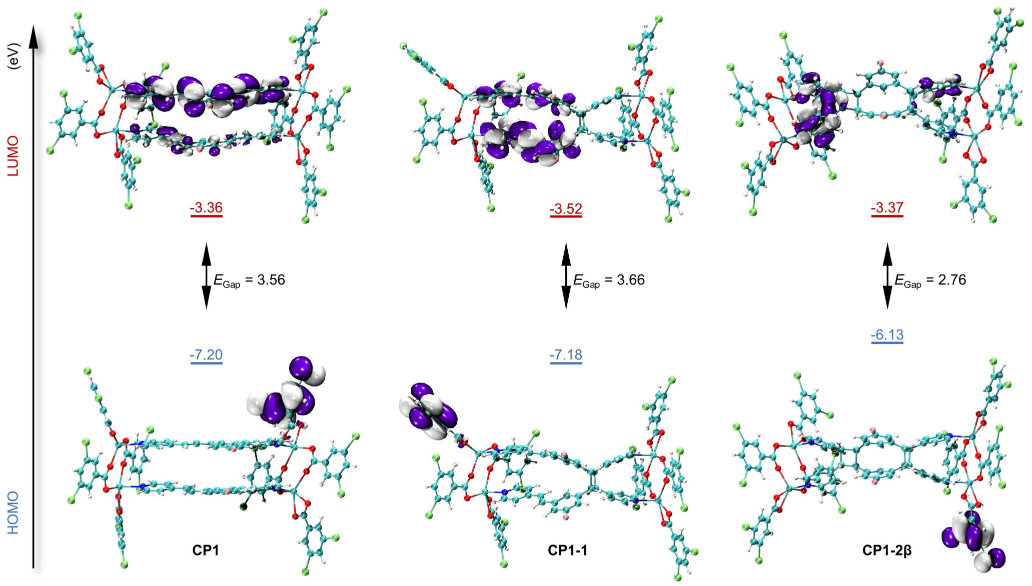

Supplementary Figure 20 | Theoretical calculation. Frontier molecular orbitals of optimized ground-state geometries of **CP1**, **CP1-1** and **CP1-2β** calculated by the sTDDFT method at the PBE0 D3 def2-TZVP level, ORCA 5.0.3 package. E_{Gap} represents calculated energy gap.

Ⅳ. Supplementary references

1. Li, W. X. et al. Post-synthetic modification of a two-dimensional metal-organic framework via photodimerization enables highly selective luminescent sensing of aluminum(III). *Inorg. Chem.* **57**, 13453 (2018).
2. Wang, M. F. et al. Controllable multiple-step configuration transformations in a thermal/photoinduced reaction. *Nat. Commun.* **13**, 2847 (2022).
3. Kearns, E. & D'Alessandro, D. M. Variable-temperature photocyclization kinetics in a metal-organic framework (MOF): a comparison of the Johnson-Mehl-Avrami-Kolmogorov and Finke-Watzky models. *Cryst. Growth Des.* **23**, 6100-6106 (2023).
4. Pandolfi, L. et al. The impact of solid solution composition on kinetics and mechanism of [2+2] photodimerizations of cinnamic acid derivatives. *CrystEngComm.* **23**, 1352-1359 (2021)
5. Huang, X. D., Jia, J. G., Kurmoo, M., Bao, S. S. & Zheng, L. M. Interplay of anthracene luminescence and dysprosium magnetism by steric control of photodimerization. *Dalton Trans.* **48**, 13769-13779 (2019).
6. Jarvis, A. G. et al. Photochemical-mediated solid-state [2+2]-cycloaddition reactions of an unsymmetrical dibenzylidene acetone (monothiophos-dba). *CrystEngComm.* **14**, 5564-5571 (2012).
7. Simone d'Agostino, S., Spinelli, F., Boanini, E., Braga, D. & Grepioni, F. Single crystal to single crystal [2+2] photoreactions in chloride and sulphate salts of 4-amino-cinnamic acid via solid-solution formation: a structural and kinetic study. *Chem. Commun.* **52**, 1899-1902 (2016).
8. Yan, B., Shi, R., Zhang, B. & Kshirsagar, T. A kinetic study of product cleavage reactions from the solid phase by a biocompatible and removable cleaving reagent, HCl. *J. Comb. Chem.* **9**, 684-689 (2007).
